# Mutational analysis of *BRCA1* and *BRCA2* genes in Peruvian families with hereditary breast and ovarian cancer

**DOI:** 10.1002/mgg3.301

**Published:** 2017-06-28

**Authors:** Jose Buleje, Maria Guevara‐Fujita, Oscar Acosta, Francia D. P. Huaman, Pierina Danos, Alexis Murillo, Joseph A. Pinto, Jhajaira M. Araujo, Alfredo Aguilar, Jaime Ponce, Carlos Vigil, Carlos Castaneda, Gabriela Calderon, Henry L. Gomez, Ricardo Fujita

**Affiliations:** ^1^ Centro de Genética y Biología Molecular Facultad de Medicina Humana Universidad de San Martín de Porres Lima Perú; ^2^ Unidad de Investigación Básica y Traslacional Oncosalud‐AUNA Lima Perú; ^3^ Unidad de la Mama Oncosalud‐AUNA Lima Perú

**Keywords:** *BRCA1* gene, *BRCA2* gene, hereditary breast and ovarian cancer syndrome, molecular analysis

## Abstract

**Background:**

Breast cancer is one of the most prevalent malignancies in the world. In Peru, breast cancer is the second cause of death among women. Five to ten percent of patients present a high genetic predisposition due to *BRCA1* and *BRCA2* germline mutations.

**Methods:**

We performed a comprehensive analysis of *BRCA1* and *BRCA2* genes by Sanger sequencing and multiplex ligation‐dependent probe amplification (MLPA) to detect large rearrangements in patients from 18 families, which met the criteria for hereditary breast cancer.

**Results:**

In this series, we found four pathogenic mutations, three previously reported (*BRCA1*: c.302‐1G>C and c.815_824dup10; *BRCA2*: c.5946delT) and a duplication of adenines in exon 15 in *BRCA1* gene (c.4647_4648dupAA, ClinVar SCV000256598.1). We also found two exonic and four intronic variants of unknown significance and 28 polymorphic variants.

**Conclusion:**

This is the first report to determine the spectrum of mutations in the *BRCA1/BRCA2* genes in Peruvian families selected by clinical and genetic criteria. The alteration rate in *BRCA1/BRCA2* with proven pathogenic mutation was 22.2% (4 out 18) and this finding could be influenced by the reduced sample size or clinical criteria. In addition, we found three known *BRCA1/BRCA2* mutations and a *BRCA1* c.4647_4648dupAA as a novel pathogenic mutation.

## Introduction

Breast cancer is one of the most common malignancies in women in the world (Karami and Mehdipour 2013) and is second to cervical cancer in Peru (Ramos and Venegas 2013). Germline mutations in *BRCA1* and *BRCA2* are the two main genes predisposing to early development of breast and ovarian tumors; they account for the majority of cases in families with high‐risk profiles, representing a 26% to 84% lifetime risk for those cancers (Malone et al. 2006). The frequency of breast and ovarian cancer families with missense mutations, short deletions, or duplications varies between 15% and 70% depending on the population studied (Thorlacius et al. 1997; Tereschenko et al. 2002; Díez et al. 2003; Caporale and Swenson 2014).

The majority of mutations in *BRCA1* and *BRCA2* (Breast Cancer Information Core, BIC database 2017) are frameshift changes resulting in nonfunctional proteins. In addition, mutations that affect intron consensus sequences at the splice and branch sites of *BRCA1* and *BRCA2* and those that create ectopic splice sites account for 15% of point mutations associated with breast and ovarian cancer (Krawczak et al. 1992; Gutierrez‐Enriquez et al. 2009). Even though almost all cases involve one single pathogenic mutation in one of these genes, there are reports of two concurrent germline mutations in the same family (Stoppa et al. 1996; Gershoni‐Baruch et al. 1997; Friedman et al. 1998; Liede et al. 1998; Moslehi et al. 2000; Ganguly et al. 2001).

More recently, the use of new methodologies has allowed the detection of large genomic alterations that involve deletions or duplications of one or more exons in *BRCA1* and *BRCA2* genes in patients who are negative for other *BRCA1/BRCA2* mutations. Those rearrangements include a gene copy number variations or deletions/duplications of exons at a frequency of 10%–20% depending on the ethnic and selection criteria of patients with hereditary breast cancer (Sluiter and van Rensburg 2011; Judkins et al. 2012). There are reports of genomic deletions and duplications mostly in the *BRCA1* gene (Woodward et al. 2005), with fewer cases involving *BRCA2* (Gutierrez‐Enriquez et al. 2007).

Genetic analysis of *BRCA1* and *BRCA2* in hereditary breast cancer families has health implications because it could offer different options to reduce the risk of developing cancer in women mutation carriers (Hernández et al. 2014).

Previous studies have shown an ancestry bias of global geographic origin for some causal mutations of different diseases, and in hereditary breast and ovarian cancer, some mutations in *BRCA1* and *BRCA2* are common to specific ethnic groups; notably, the mutations 185delAG in Ashkenazi Jews and 943ins10 in African‐Americans and Bahamians (Hall et al. 2009; Donenberg et al. 2011). The Peruvian population is currently over 30 million and has an estimated average indigenous genetic background of 70%, with the remaining European, African, and Asian ancestry fluctuating in the 5%–15% range, depending on the geographical origin (Elhaik et al. 2013; Sandoval et al. 2013). In general, the Peruvian and South American indigeneous populations have been the subject of very few genetic studies, mostly represented in the generic nominations of Latinos or Hispanic (Bryc et al. 2010). The only report on *BRCA1* and *BRCA2* mutations causing breast and ovarian cancer specifically in Hispanos was conducted using a custom‐made chip “*Hispanel*” developed at the City of Hope Comprehensive Cancer Center, Duarte, California, USA (Villarreal‐Garza et al. 2015).

We wanted to examine whether if the frequency of *BRCA1* and *BRCA2* mutations in Peruvian patients has the same pattern as other Hispanic populations and whether novel mutations of possible autochthonous South American origin exist.

## Materials and Methods

### Subjects

Blood samples were collected from 28 patients belonging to 18 families with a high risk for hereditary breast cancer. Patients were identified at Oncosalud‐AUNA clinic, a comprehensive cancer center in Peru, and referred for genetic analysis at the Centro de Genética y Biología Molecular of the Universidad de San Martín de Porres to gather information about pedigree and other cancer occurrences in the family, and to take venous blood for DNA. The selection of high‐risk families was made following the National Comprehensive Cancer Network (NCCN) criteria for hereditary breast and ovarian cancer. The study protocol and informed consent form was approved by the international review board of the Universidad de San Martín de Porres (IRB00003251‐FWA0015320). All patients signed an informed consent and were interviewed by a genetic counselor for medical, family, and lifestyle history.

### Genomic DNA isolation and amplification

Genomic DNA was extracted from peripheral blood cells using the classical salting‐out extraction procedure (Miller et al. 1988), with some modifications, and was quantified using a Qubit^TM^ Fluorometer (Invitrogen^TM^, Carlsbad, CA, USA). Sixty‐six pairs of primers were used to amplify all exons and intron flanking regions of *BRCA1* and *BRCA2* genes (Rubio Rodrigo 2008). The final volume of reaction was 25 μL containing 50 ng of DNA, 10 pmol of each primer, 0.5 U of Taq polymerase (Thermo Fisher Scientific, Boston, MA, USA), 0.2 mm dNTPs, 1.5 mm MgCl_2,_ and 1× buffer. PCR was performed in a Veriti^®^ Thermocycler (Applied Biosystems^®^, Foster City, CA, USA) with initial denaturation at 95°C for 5 min, followed by 35 cycles at 95°C for 45 sec, specific annealing temperature for 1 min, 72°C for 2 min, and the final elongation step at 72°C for 10 min.

### RNA extraction and RT‐PCR

Total RNA was obtained from peripheral‐blood leukocytes of patients and controls using TRIzol reagent (Invitrogen^TM^, Carlsbad, CA, USA) according to the manufacturer's protocol. After quantification with QubitTM Fluorometer (Invitrogen^TM^, Carlsbad, CA, USA), one microgram of total RNA was reverse transcribed using QuantiTect Reverse Transcription Kit (Qiagen^®^, Hilden, Germany) according to the manufacturers’ protocols. Primers used to amplify the BRCA1 coding region from exon 6 through exon 9 were 5′‐TCAGCTTGACACAGGTTTGG‐3′ and 5′‐CTTGATCTCCCACACTGCAA‐3′, forward and reverse, respectively. This was performed to check c.302‐1G>C in the carrier and three normal controls.

### Quantitative real‐time PCR

In order to confirm the increased copy number of BRCA1 exon 7 after a MLPA screening, primers were designed to generate an amplicon of 54 pb for qPCR analysis using QuantiTect SYBR Green (Qiagen^®^, Hilden, Germany). Four different control DNA and two patients (CM03 and CM23) with copy number variations were analyzed. Calibration curve was constructed using serially diluted human control DNA (6, 3, 1.5, 0.5 ng/μL) from Epitect PCR Control DNA Set and MPL exon 10 as a normalizer gene. Quantification of the altered exon was assessed in patients and control DNA by normalizing all Ct values with the calibration curve and generating a [BRCA1]/[MPL] ratio. The ratio values were compared to determine the copy number variation between samples.

### DNA sequencing

PCR amplicons were purified using a GeneJET PCR Purification Kit (Thermo Fisher Scientific, Boston, MA, USA) and sequenced in both directions using the BigDye Terminator version 3.1 Cycle Sequencing Kit and the ABI PRISM 3500 Genetic Analyzer (Applied Biosystems^®^, Foster City, CA, USA). The generated DNA sequences were analyzed using Sequencing Analysis Software 5.1 (Applied Biosystems^®^, Foster City, CA, USA) and then aligned using the Basic Local Alignment Search Tool (BLAST, available from: http://blast.ncbi.nlm.nih.gov/Blast.cgi). All mutations were identified by making comparisons with reference sequences from the National Center for Biotechnology Information database of genetic variation. The mutations were described by the guidelines proposed by the Human Genome Variation Society (HGVS) site and were referred to the cDNA sequence of *BRCA1* (NM_007294.3) and *BRCA2* (NM_000059.3).

### Multiplex ligation‐dependent probe amplification (MLPA)

The MLPA technique was used to detect changes in the number of copies of the *BRCA1* and *BRCA2* genes or exons using SALSA MLPA probemix *BRCA1* P002‐C2, probemix *BRCA2* P045/CHEK2, and SALSA MLPA reagent kit‐FAM (MRC‐Holland, Amsterdam, the Netherlands).

The procedure was carried out as suggested by the MRC‐Holland protocol (MLPA‐DNA protocol version MDP‐V003; MRC‐Holland 2013), which consisted of four steps: (1) DNA denaturation (30–50 ng) at 98°C for 5 min, (2) hybridization with a specific probemix for 16 h at 60°C, (3) ligation at 54°C for 30 min, (4) inactivation ligase at 98°C for 5 min, (5) and amplification of fragments.

For capillary electrophoresis, a mixture of 1 uL product amplification, 0.2 uL weight marker (LIZ 500), and 9 uL of HiDi formamide, denatured for 3 min at 90°C, was used for each patient on an ABI 3500 Genetic Analyzer using a 50 cm capillary with POP7 resine. Amplification was performed with a 1.6‐kV injection voltage and a 15‐sec injection time.

Data were analyzed using the Coffalyser software, as proposed by MRC‐Holland (Amsterdam, the Netherlands) in the MLPA General Protocol version MDP‐V003. This analysis comprised three stages: the record of fragments followed by a comparative analysis and analysis of Coffalyser score (CAS). If the samples have both optimum parameters, the program uses the ratio of fluorescence (DQ) to quantify the number of copies present in each fragment.

### Population‐based study by high‐resolution melting (HRM)

The frequency in the control populations of novel variants in *BRCA1* (p.C47F) and *BRCA2* (p.R155K) were evaluated in 100 control individual DNAs (200 chromosomes) by HRM of the amplicons containing the variants. PCR reactions were performed in 12.5 ul final volume using the Type‐it HRM PCR Kit (Qiagen^®^, Hilden, Germany), free nuclease water, and 15 ng DNA using a Rotor Gene Q 5‐plex HRM (Qiagen^®^, Hilden, Germany) provided with the Rotor‐Gene Q Series Software Version 2.3.1. The primer mix for both cases was used at 0.7 uM. PCR conditions consisted of a two‐step program with a denaturing step at 95°C for 10 sec and an annealing‐extension step at 55°C (p.C47F) and 60°C (p.R155K) for 30 sec. A preliminary melting analysis was performed previous to HRM by taking continuous fluorescent readings from 60 to 90°C, rising by 0.5°C in each step. Once the critical temperature ranges were identified, HRM was performed in the following conditions: rising by 0.05°C in each step, from 72 to 78°C for the p.C47F assay, and from 69.5 to 78.5°C for the p.R155K assay, which were thus acquired and registered in the Rotor Gene HRM channel.

The melting curve analysis was performed by shifting the temperature axis around the melting temperature detected for each amplicon. Genotyping was accomplished using positive controls for the mutation and wild type genotypes, both confirmed previously by Sanger sequencing. Wild type and mutant genotypes were considered above 80% confidence by normalization of melting curves. Finally, the difference plot helped to cluster the samples into groups. The PCR products were confirmed by agarose gel electrophoresis ensuring the presence of a single PCR product with the expected size.

### Bioinformatics predictions of variants

To determine the functional impact of the variants of uncertain significance, we used the on‐line programs PolyPhen‐2 (http://genetics.bwh.harvard.edu/pph2/), SIFT (http://sift.jcvi.org/), and Align‐GVGD (http://agvgd.iarc.fr/agvgd_input.php). These prediction programs are based on observed residue substitutions in homologous proteins of different species. Additionally, for variants of uncertain significance we used ESEfinder software version 3.0 (http://rulai.cshl.edu/cgi-bin/tools/ESE3/esefinder.cgi) to predict whether they affect an exonic splicing enhancer.

## Results

All families fulfilled the selection criteria for hereditary breast and ovarian cancer established in the study. We screened 28 individuals belonging to 18 families to obtain the presence of point mutations and large genomic rearrangements in *BRCA1* and *BRCA2* genes. When a presumed pathogenic mutation was identified in a patient, we screened some affected relatives for the presence of the particular mutation. MLPA analysis was performed in all probands.

### Pathogenic mutations

In our series, we identified four family carriers (22%) of germline mutations in *BRCA1* and *BRCA2* genes. Table 1 shows the features of families carrying a germline mutation in *BRCA1* and *BRCA2* and Table 2 summarizes the list of pathogenic mutations identified in four families.

**Table 1:** Features of families carrying a germline mutation in BRCA1 and BRCA2

Family ID	Clinical manifestations (age of onset)	Family history
Fam 3	Breast cancer (40)	Father with prostate cancer, half‐sister with bilateral breast cancer at age 34, grandfather with prostate cancer, and one second‐degree relative with breast cancer at age 50.
Fam 12	Breast cancer (45)	Father with prostate cancer at age 75, aunt with breast cancer, two cousins with breast cancer, grandfather with lung cancer, and two second‐degree relatives with breast cancer.
Fam 13	Breast cancer (36)	Mother with ovarian cancer at 45, aunt with breast cancer at 45, grandmother with breast cancer at 68, three of mother's half‐sisters with breast cancer at ages 29, 38, and 50, and one cousin with testicular cancer.
Fam 17	Breast and ovarian cancer (46)	Mother with breast cancer, two aunts with breast cancer, one aunt and one grand uncle with stomach cancer, one aunt with ovarian cancer, and one uncle with lung cancer.

**Table 2:** Pathogenic germline mutations detected in *BRCA1* and *BRCA2* genes

Family ID	Gene	Location	Nucleotide change	Protein effect	dbSNP	Submission number (ClinVar)
Fam 12	BRCA1	Intron 6	c.302‐1G>C	–	rs80358116	SCV000263345.1
Fam 17	BRCA1	Exon 11	c.815_824dup10	p.T276Afs*14	rs387906563	SCV000263346.1
Fam 13	BRCA1	Exon 15	**c.4647_4648dupAA**	p.T1550Kfs*10	**rs869025213**	**SCV000256598.1**
Fam 3	BRCA2	Exon 11	c.5946delT	p.S1982Rfs*22	rs80359550	SCV000263344.1

Bold annotation indicates a novel mutation found in our study.

**Figure 1:**
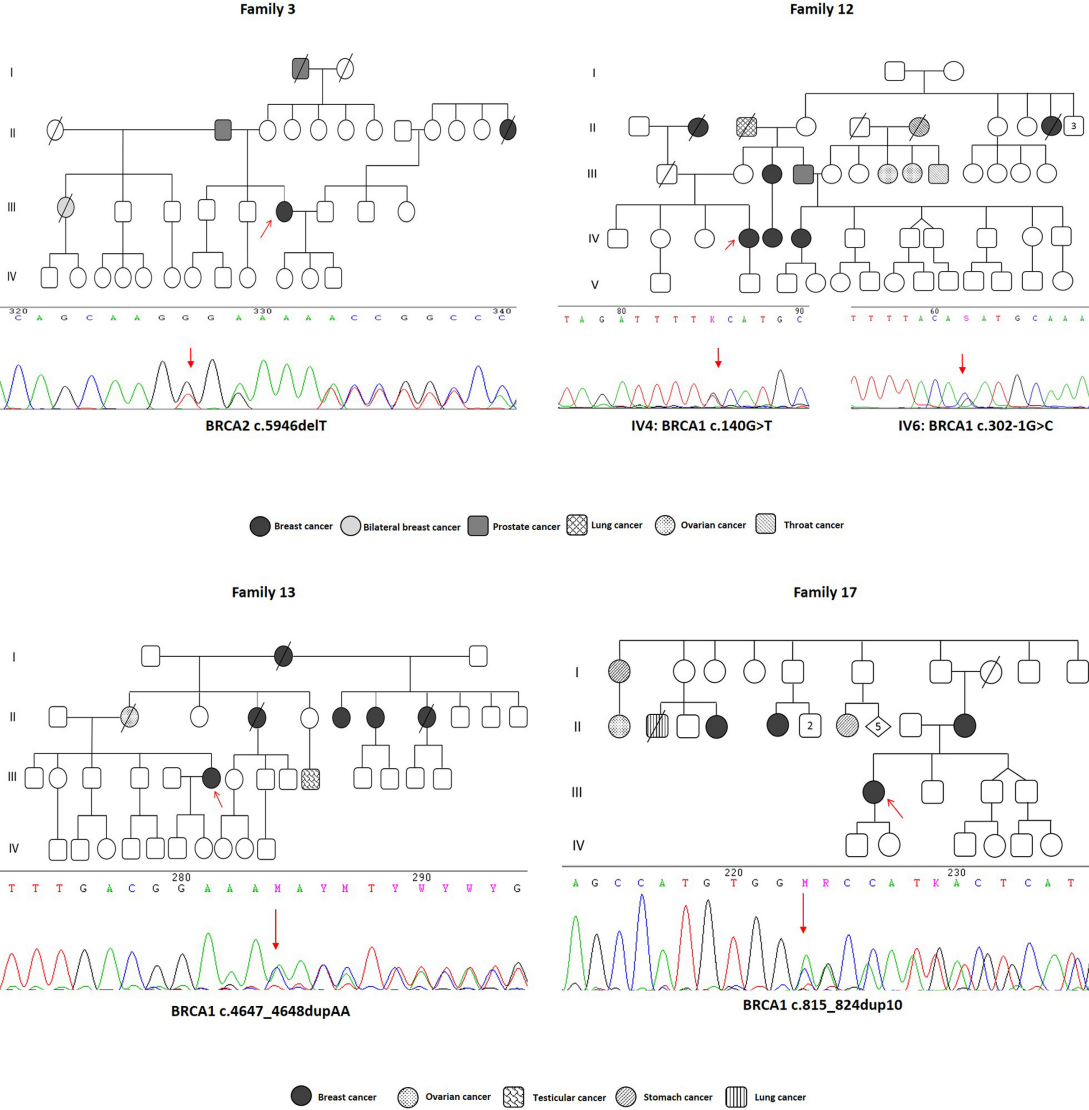
Genealogy and Sanger sequencing of families with pathogenic germline mutations. Family 3 presented an Ashkenazi mutation (c.5946delT) in BRCA2. Family 12 presented a splice exon skipping alteration (c.302‐1G>C) and missense mutation (c.140G>T) in intron 6 and exon 5 of BRCA1, respectively. Family 13 presented a duplication of two adenines (c.4647_4648dupAA) in exon 15 of BRCA1, and family 17 presented a duplication of 10 nucleotides (c.815_824dup10) in exon 11 of BRCA1. Arrow indicates proband and mutation position.

Splice site change mutation c.302‐1G>C, located in intron 6 of *BRCA1*, was detected in family 12 in a maternal first‐degree cousin of the proband with bilateral breast cancer at age 51 (Fig. 1). Proband with breast cancer at the age of 36, has an extended maternal family with a high incidence of cancer showing three of eight individuals in generation II and five out of 12 in generation III and at least two maternal first‐degree cousins with breast cancer carry the c.302‐1G>C mutation. The *BRCA1* c.302‐1G>C mutation has been reported in the ClinVar database as pathogenic. At the same nucleotide, *BRCA1* c.302‐1, other variants were also reported as pathogenic (c.302‐1G>A; c.302‐1G>T) (Gutierrez‐Enriquez et al. 2009). The proband did not show that splice mutation but another one (see below p.C47F in the VUS section).

Another mutation, a duplication of 10 bases c.815_824dup10, located in exon 11 of *BRCA1*, was detected in a patient diagnosed with breast cancer at age 46 from family 17. This mutation leads to a frameshift and a spurious stop codon of 14 amino acids further down (p.T276Afs*14). The patient's mother and two aunts had breast cancer, one aunt and one grand uncle had stomach cancer, one aunt had ovarian cancer, and one uncle had lung cancer (Fig. 1).

We identified c.5946delT in *BRCA2* (BIC: 6174delT), a mutation which is known to be a founder mutation in Ashkenazi Jewish ancestry. This mutation was reported in a patient in family 3, who was diagnosed with breast cancer at age 40. Her paternal half‐sister had bilateral breast cancer at age 34, her father and grandfather had prostate cancer, and a second‐degree relative had breast cancer at age 50 (Fig. 1).

In addition, a novel mutation was found in this study, c.4647_4648dupAA (Submission number SCV000256598.1; rs869025213), which is located in exon 15 of *BRCA1*. Its putative product results in a frameshift and the termination of protein translation for 10 residues further down (p.T1550Kfs*10). This mutation was identified in family 13 and the proband was diagnosed with breast cancer at 36 years. The proband's mother got ovarian cancer at age 45, her maternal aunt had breast cancer at age 45, her maternal grandmother had breast cancer at age 68, three of the mother's half‐sisters had breast cancer at ages 29, 38, and 50, and one cousin had testicular cancer (Fig. 1).

### Gene dosage alterations

**Figure 2:**
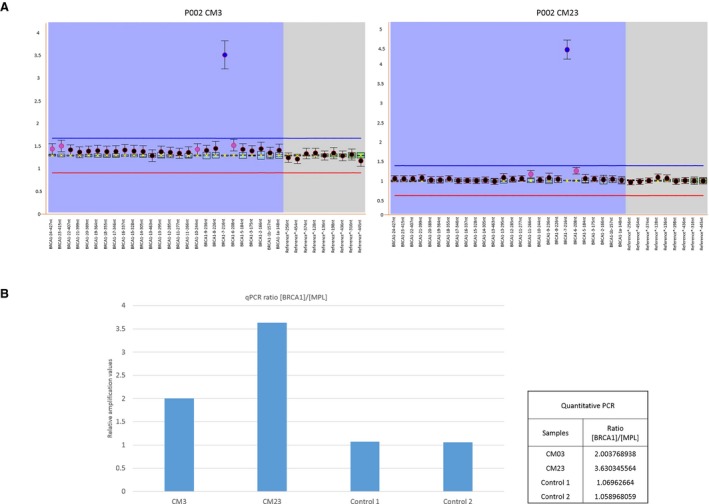
(A) MLPA results for BRCA genes. The left electropherogram shows an exon 7 amplification (ratio 2.74) in a BRCA1 patient (CM03), which presents an Ashkenazi mutation (c.5946delT) in BRCA2. The right electropherogram shows an amplification of exon 7 (ratio 3.62) in another BRCA1 patient (CM23) with familial breast cancer. We evaluated the second amplification in an affected relative but she did not present the alteration. We used Coffalyser.Net software for analysis. Both figures show BRCA1 exons on the *X*‐axis and intranormalized ratio on *Y*‐axis, respectively. All assays were performed three times. (B) qPCR to confirm results of MLPA.

The 18 families were screened for the presence of large genomic rearrangements using MLPA, which were found in two patients. An amplification in a copy number of exon 7 of *BRCA1* was found (Fig. 2) in a patient (CM03, family 2) diagnosed with breast cancer at age 40. Her sister also had breast cancer at age 34 and died. In addition, her father and grandfather were diagnosed with prostate cancer. Another patient unrelated to this family also had a rearrangement of exon 7 of *BRCA1* (CM23, family 14), and she was diagnosed with bilateral breast cancer at age 60. This rearrangement was analyzed in her sister with breast cancer at age 67, but that amplification was not present. Both rearrangements were analyzed for triplicate in independent assays for confirmation.

MLPA amplification results were corroborated by performing a quantitative PCR (Fig. 2) using primers flanking exon 7 of *BRCA1* and primers designed for *MPL* exon 10 as a normal control (2X) endogenous gene. Extra copies for exon 7 *BRCA1* were detected for both patients by qPCR relative amplification. Values shown that both patients have an abnormal ratio compared with controls, reflecting the presence of extra copies.

### Variants of uncertain significance (VUS)

**Figure 3:**
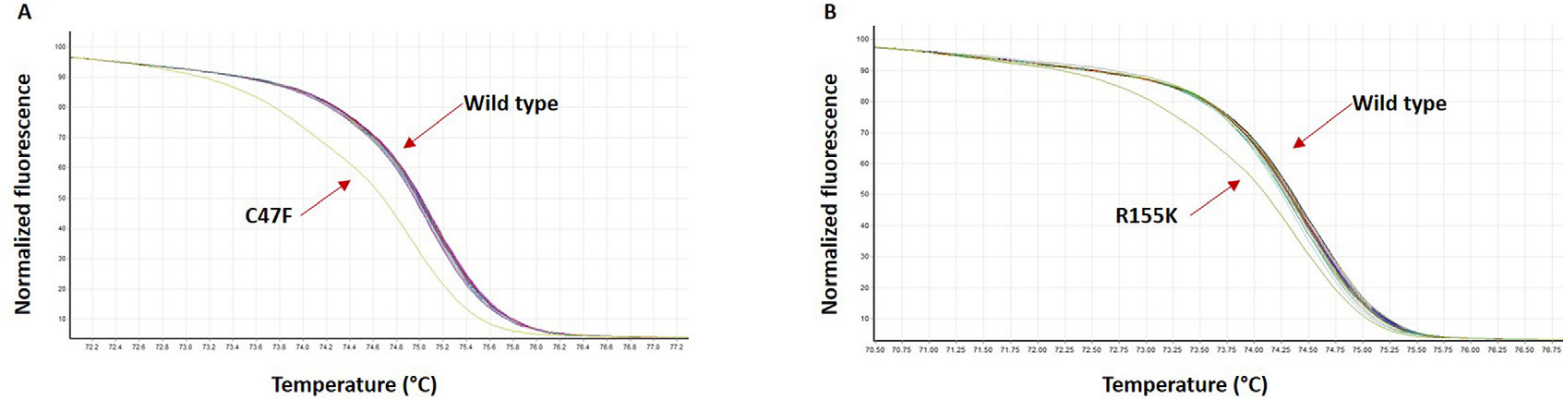
Population‐based study by HRM analysis. The figure shows the different fluorescence‐normalized HRM plots for each variant of unknown significance. Each curve represents the amplicon from a different individual's DNA sample. Panel (A) BRCA1 exon 5: p.C47F; and Panel (B) BRCA2 exon 5: p.R155K. The analysis of variants was compared with 100 control samples.

Two exonic variants of unknown significance were identified, one in *BRCA1* and the other in *BRCA2* (Table 3). We reported a VUS in exon 5, c.140G>T, a missense, which generated a change from cysteine to phenylalanine at codon 47 (p.C47F; submission number SCV000282660.1; rs80357150) in the RING domain of the protein. According to the Universal Mutation Database software (http://www.umd.be/), p.C47F is pathogenic. The in silico predictors SIFT, CGCD, and ESEfinder suggest its pathogenicity that has been proven by functional assays (Donenberg et al. 2011). The proband of family 13 bears that mutation, and she has a maternal first cousin with c.302‐1G>C. The paternal branch of the proband family had cases of cancer including her father with prostate cancer at age 75, a paternal aunt and two cousins with breast cancer, a grandfather with lung cancer, and two second‐degree relatives with breast cancer. The other VUS was a novel *BRCA2* missense variant c.464G>A in exon 5, which generated a change from arginine to lysine at codon 155 (p.R155K; submission number SCV000282661.1; rs377639990; Table 3). When analyzed by the predictors, it is determined as “tolerated” by SIFT analysis, with a CGCD score of 25, and no disruption reported by ESEfinder, indicating that this mutation shows low probabilities of pathogenicity. To clarify the function of these variants C47F and R155K, we performed a population‐based study using DNA from 100 nonselected, normal adult Peruvian controls (200 chromosomes), who have not developed cancer, of all ages and both sexes. These variants were analyzed using HRM (Fig. 3), and neither was found in the control population, indicating that both variants remain uncommon in the general population.

**Table 3:** List of exonic variants of uncertain significance (VUS) and in silico analysis

Gene	Nucleotide change	Protein effect	Location	Functional domain	Database				In silico				Functional assay	Submission number (ClinVar)
					UMD	BIC	ClinVar	dbSNP	Polyphen	SIFT	Align GCGD	ESEfinder		
**BRCA1**	c.140G>T	C47F	Exon 5	Ring	Pathogenic	unknown	VUS	rs80357150	Possibly	Affect	C65	ESE disruption (SRSF6)	Pathogenic (Donenberg et al. 2011)	SCV000282660
**BRCA2**	**c.464G>A**	**R155K**	**Exon 5**	–	–	–	–	**rs377639990**	Probably	Tolerated	C25	No effect	–	SCV000282661

Polyphen: variant benign, Possibly damaging and Probably damaging; SIFT: Variant tolerated (benign) or Affect protein function; Align GVGD: C0 (Less likely to interfere in protein function), C15, C25, C35, C45, C55, C65 (More likely to interfere in protein function); ESEFinder: ESE (Exonic Splicing Enhancer), SRSF6 (Serine/arginine‐rich splicing factor 6). Bold annotation indicates a novel mutation found in our study.

In addition, we identified four intronic variants of unknown significance (three of which were novel mutations c.442‐9A>T in intron 7, c.5074+28T>A in intron 17, and c.5193+79A>G in intron 1 as well as a new previously reported c.548‐64delT,) in *BRCA1* and tried to characterize them by in silico analysis (Table 4).

**Table 4:** Intronic variants of uncertain significance (VUS)

Nucleotide change	Location	No. of patients with variant	dbSNP	ESEfinder
c.442‐9A>T	Intron 7	2	**Novel**	No effect
c.548‐64delT	Intron 8	2	rs273902772	No effect
c.5074+28T>A	Intron 17	7	**Novel**	Add ESE (SRSF2)
c.5193+79A>G	Intron 19	1	**Novel**	Loss ESE (SRSF5)

Bold annotation indicates a novel mutation found in our study.

### Polymorphisms

We found 28 polymorphic variants (in exons or introns) in *BRCA1* and *BRCA2* genes previously reported at the dbSNP and UMD database: nine silent mutations in the coding sequence, eight intronic variants, ten missense variants, and the c.1‐26G>A variant on the 5′ UTR region (Table 5).

**Table 5:** Polymorphisms in *BRCA1* and *BRCA2* genes

Gene	Location	Nucleotide change	Protein effect	No. patients with the variant	dbSNP
BRCA1	Missense variations				
	Exon 11	c.1067A>G	p.Q356R	2	rs1799950
	Exon 11	c.2612C>T	p.P871L	8	rs799917
	Exon 11	c.3113A>G	p.E1038G	6	rs16941
	Exon 11	c.3548A>G	p.K1183R	12	rs16942
	Exon 15	c.4535G>T	p.S1512I	1	rs1800744
	Exon 16	c.4837A>G	p.S1613G	12	rs1799966
	Synonyms				
	Exon 11	c.2082C>T	p.S694S	10	rs1799949
	Exon 11	c.2311T>C	p.L771L	11	rs16940
	Exon 13	c.4308T>C	p.S1436S	14	rs1060915
	Intronic variations				
	Intron 7	c.441+36delCTT		7	a
	Intron 7	c.441+36del14		7	a
	Intron 7	c.441+51delT		5	a
	Intron 7	c.442‐34C>T		2	rs799923
	Intron 14	c.4485‐63C>G		6	rs273900734
	Intron 18	c.5152+66G>A		11	rs3092994
BRCA2	Missense variations				
	Exon 10	c.865A>C	p.N289H	5	rs766173
	Exon 11	c.2971A>G	p.N991D	4	rs1799944
	Exon 11	c.5744C>T	p.T1915M	1	rs4987117
	Exon 14	c.7397C>T	p.A2466V	1	rs169547
	Synonyms				
	Exon 10	c.1365A>G	p.S455S	4	rs1801439
	Exon 11	c.2229T>C	p.H743H	5	rs1801499
	Exon 11	c.3396A>G	p.K1132K	5	rs1801406
	Exon 11	c.3807T>C	p.V1269V	3	rs543304
	Exon 11	c.4563A>G	p.L1521L	17	rs206075
	Exon 14	c.7242A>G	p.S2414S	4	rs1799955
	Intronic variations				
	Intron 8	c.681+56C>T		6	rs2126042
	Intron 10	c.1909+22delT		17	rs587780561
	UTR variations				
	5′UTR	c.1‐26G>A		3	rs1799943

a Only reported in UMD database.

## Discussion

As familial cancer history increases the risk of malignancy in individuals, it also provides a reason for preventive measures such as lifestyles changes, more frequent screenings, chemoprevention, prophylactic surgery, and genetic detection, when possible. Breast and ovarian cancer caused by mutations in *BRCA1* and *BRCA2* have been identified as the most common type of inherited cancer (Scheuner et al. 2010), and the founder effect is observed in populations like of Ashkenazi or Mexican Ancestry (Díez et al. 2003; Weitzel et al. 2005; Hall et al. 2009; Villarreal‐Garza et al. 2015; Dutil et al. 2015). To our knowledge, the only study on *BRCA1* and *BRCA2* genes in Peruvian populations was performed with a panel chip, “Hispanel,” of 114 recurrent mutations that are claimed to represent 80% of Hispanic BRCA1 and BRCA2 mutations (Abugattas et al. 2014). In that study, 266 unselected nonrelated breast cancer patients showed 13 deleterious mutations (11 *BRCA1* and 2 *BRCA2*), including *BRCA1* 185delAG present in seven patients, *BRCA1* 2080delA in two patients, and *BRCA2* 3036del4 in two patients.

South Americans in general, and specifically Peruvian hereditary breast cancer populations, are not well characterized with mutations in *BRCA1* and *BRCA2* genes, and a founder effect has not been identified. We studied a group of 18 families with hereditary breast and ovarian cancer by family history and age of presentation criteria (<55 years old) and performed complete exons and partial intron sequencing of *BRCA1/2* genes and screening for large rearrangements by MLPA. We used both methods to avoid losing information on mutations or underestimating the prevalence and diversity of *BRCA* mutations in our population.

### Pathogenic mutations

**Figure 4:**
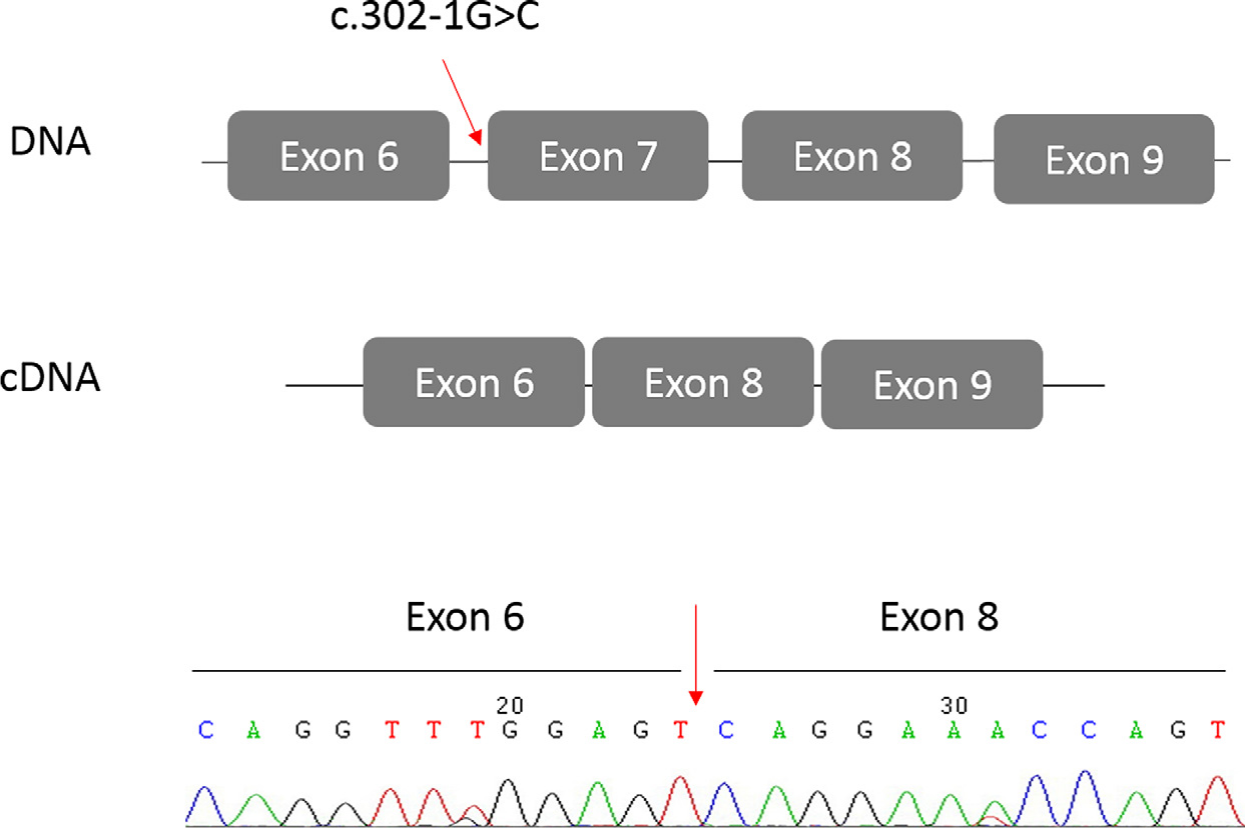
Partial sequence of the RT‐PCR product of mutated allele from a BRCA1 c.302‐1G>C. This alteration causes skipping of exon 7, and the appearance of a premature stop codon (p.Q141EfsX8).

One of the main types of alternative splicing is alternative acceptor site selection (Kim et al. 2008), and the splice acceptor mutation c.302‐1G>C (at the canonical intronic acceptor splicing site AG) found in the proband of family 12 has been previously reported as pathogenic (Breast Cancer Information Core (BIC), 2017), although its functional mechanism has not been explored. However, a similar missense c.302‐1G>A creates a new alternative acceptor site out of frame in exon 7 of *BRCA1* adding 14 spurious residues and a predicted stop codon at residue 115 (p.Tyr101SerfsX15) (Gutierrez‐Enriquez et al. 2009), which can be analyzed by cDNA sequencing. Our analysis of c.302‐1G>C cDNA products (Fig. 4) demonstrates that this intronic substitution generate a transcript lacking exon 7, with the inclusion of seven new amino acids (141^147) in the *BRCA1* protein and the appearance of premature stop codon in position 148 (p.Q141EfsX8). This results support their pathogenic effect. This mutation was present in the maternal branch of the proband, where at least 10 individuals had cancer (six with breast or ovarian, two with throat, one with prostate, and one with lung cancer). That mutation was also found in another maternal first cousin with breast cancer in a commercial service. Unexpectedly, that splice mutation present in the maternal branch of the proband was not found in her (cancer diagnosed at age 43) or her healthy mother (age 71), but rather a VUS missense p.C47F was found. This mutation was absent in her mother, and the lack of information from the father and paternal relatives precluded us from further analysis in this branch of her family. That concurrence of two events of mutation in a proband family merits a short reflection in the diagnosis because the proband approached for corroboration of the diagnosis for the high risk of bearing the c.302‐1G>C from her maternal family and the negative result prompted us to conduct further analysis to find the missense p.C47F in exon 5 of *BRCA1*.

The novel mutation c.4647_4648dupAA found in family 13 lies outside the functional domain of *BRCA1*, but the frameshift and the stop codon created (10 residues further down) in exon 15 generated the loss of the C‐terminal BRCT repeat domain, which mediates protein–protein interactions (cell cycle checkpoint for DNA damage) (Au and Henderson 2005), and the truncation of the BRCT domain greatly impairs the stability and nuclear localization of the *BRCA1* protein (Nelson and Holt 2010).

The *BRCA1* c.815_824dup10 mutation in family 17 is the most frequent pathogenic variant reported in African‐Americans (Scheuner et al. 2010). There are also reports about this mutation in Mexican families (Weitzel et al. 2005), patients of Latin American/Caribbean background in the BIC database (Breast Cancer Information Core (BIC), 2017), and in unselected breast cancer patients from Peru (Abugattas et al. 2014).

The c.5946delT (BIC: 6174delT) in *BRCA2* found in family 3 is a well characterized founder mutation in Ashkenazi Jews and is present in about 1.52% of the Ashkenazi Jewish. The average risk of breast cancer by the age of 70 for Ashkenazi carriers of the c.5946delT mutation is higher than the risk of ovarian cancer, 43% and 20%, respectively (Janavicius 2010).

Concerning the Hispanel (Hispanic panel), made mostly with samples of indigenous and mestizos mainly from Mexican and Mexican‐American origin (31), we only detected one common mutation, c.5946delT (BIC: 6174delT), in *BRCA2* of Ashkenazi ancestry. This finding raises concerns about the utility of the panel when applied to Hispanic populations that are different to the original population source and points out the need for a wider and more representative sample in those populations.

### Variants of uncertain significance (VUS)

**Figure 5:**
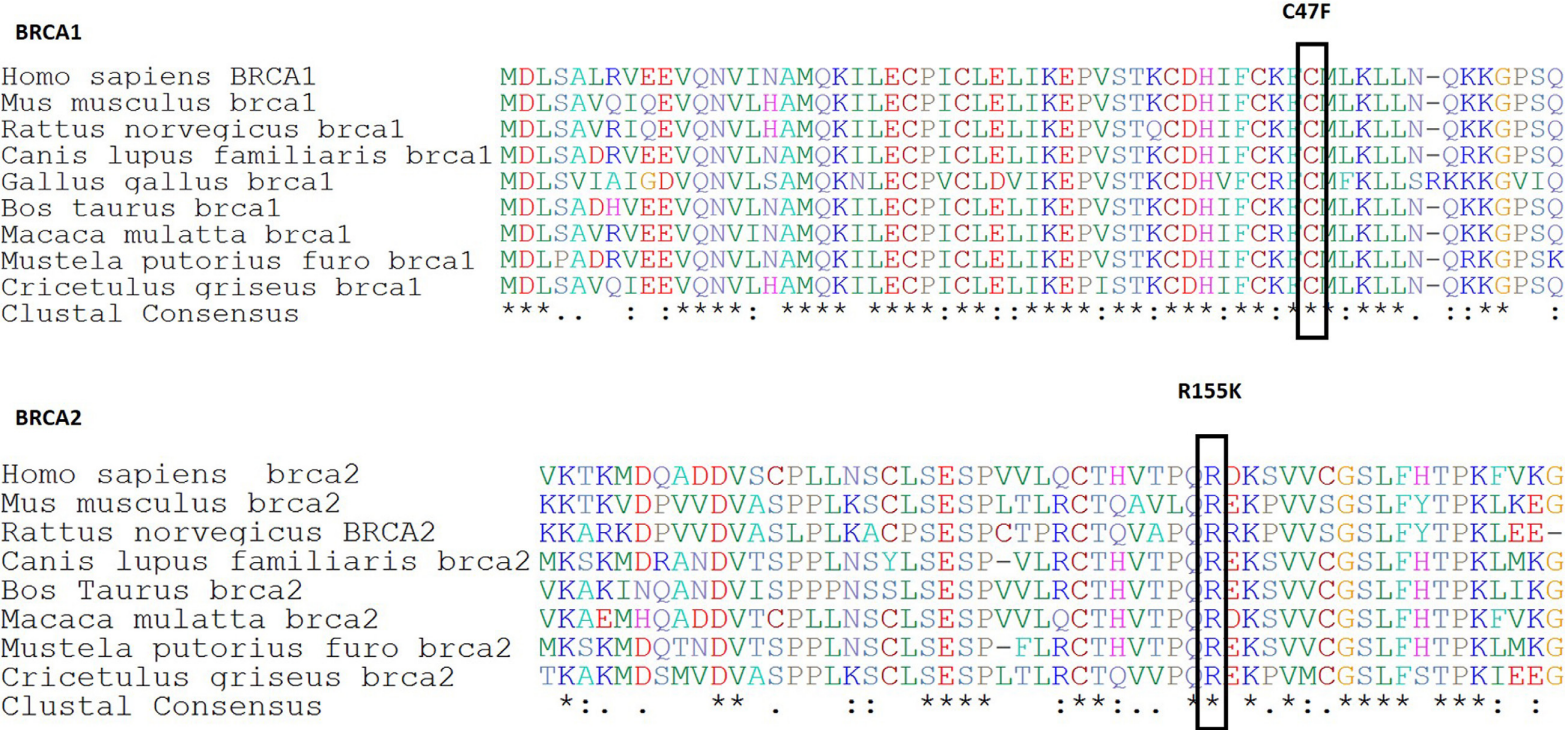
Multiple alignments using ClustalW and amino acid conservation of two missense variations: c.140G>T (p.C47F) in BRCA1 and c.464G>A (p.R155K) in BRCA2 across several species. Letter in the box denotes amino acids substituted. Cysteine at codon 47 in BRCA1 and arginine at codon 155 in BRCA2 are conserved across different species. Asterisk denotes conserved amino acid.

The variants with unknown pathogenic potential are termed unclassified variants and account for approximately 50% of the variants detected in *BRCA1* and *BRCA2* (van der Groep et al. 2011). The *BRCA1* missense mutation c.140G>T, which lies inside the RING domain, generates a change from cysteine to phenylalanine at codon 47 (p.C47F). It has been reported in the BIC database and ClinVar as of unknown significance, although this cysteine is very well conserved across species (Fig. 5). Bioinformatic tools (Polyphen and SIFT) and functional assays (Millot et al. 2011; Meenakumari and Rajkumar 2012) indicate C47F may alter protein function. ESEfinder software predicted an ESE disruption (SRSF6), whereas Align GCGD predicted a high‐risk estimate for this missense mutation.

The *BRCA2* missense mutation R155K is a novel mutation that was not registered in the BIC, ClinVar, or LOVD records. Arginine at position 155 is highly conserved across species, although Polyphen and SIFT software give contradictory predictions of probably damaging or tolerated, respectively. ESEfinder software reported no effect, and Align GCGD reported a low risk estimate for this missense mutation.

Our analysis of two VUS by population‐based study helps to clarify the possible effects of C47F and R155K, but is necessary to complete the analysis with co‐segregation studies.

The clinical utility of VUS is limited by the lack of knowledge about its pathogenicity. A subset of the VUS is located in intronic sequences and these variants may play a role in the regulation of pre‐mRNA splicing and can be deleterious (Wong‐Brown et al. 2013). Some splice site prediction programs (SSPPs) have been developed to predict the possible effect of a variant on RNA splicing. In our case, ESEfinder (v.3.0) software demonstrated a significant variation in the branch site between the wild type and the mutant sequence in c.5074+28T>A (new site SRSF2) and c.5193+79A>G (lost site SRSF5) mutations, but no variation in c.442‐9A>T and c.548‐64delT (Table 4). It is essential to establish the effect of these variants on RNA splicing by molecular RNA analysis to assess if they are benign or pathogenic.

### Polymorphic variants

We have found sequences reported as polymorphic at both *BRCA1* and *BRCA2*, presumably with no pathogenic effect (Domchek and Greenberg 2009; Jaure et al. 2015) (Table 4). Many *BRCA1* single nucleotide polymorphisms (SNPs) result in amino acid changes. Yet these polymorphisms, with the exception of Q356R and D693N, are in significant linkage disequilibrium (LD) and are inherited as part of a shared haplotype (Freedman et al. 2005). In *BRCA1*, the polymorphic variants S1613G in exon 16 and P871L accompanying E1038G in exon 11 constitute the most common single nucleotide polymorphism in the *BRCA1* gene, and they had been reported in different populations (India, Greece, Malaysia, Sri Lanka, Turkey, and Italy) (Karami and Mehdipour 2013). There are some reports about variants in *BRCA1* with protective effect; for example, the lack of K1183R variant in exon 11 of the *BRCA1* gene increases the risk of breast cancer, which could be considered protective polymorphism (Karami and Mehdipour 2013). Finally, variant S1512I is not an important functional domain, and its allelic frequency is similar in controls and patients (Deffenbaugh et al. 2002; Phelan et al. 2002).

In *BRCA2*, the very common variants N289H, N991D, T1915M, and A2466V are neutral variants of negligible functional and clinical significance, and the observed relative frequencies of these variants in unilateral and bilateral breast cancer are consistent with the hypothesis that these variants do not affect the risk of breast cancer (Freedman et al. 2004; Borg et al. 2010). However, Debniak et al. demonstrated that the common variant N991D is linked with an increased malignant melanoma risk (Debniak et al. 2008; Bougie and Weberpals 2011). Finally, the 5′ UTR polymorphism c.1‐26G>A (rs1799943) of *BRCA2* is considered benign (ClinVar), even though Gochhait et al. reported that this polymorphism might regulate the expression of *BRCA2*, albeit not at a transcriptional level, but at a posttranscriptional level affecting the translational efficiency (Gochhait et al. 2007).

### Gene dosage alterations

Detection of rearrangements in the *BRCA* genes is essential because, in some populations, the prevalence of large deletions or duplications is substantial. Such prevalence comprises 18% of Jewish families (Palma et al. 2008), 10% of Asian families (Lim et al. 2007), and between 1.7% and 5.7% of German families (Preisler‐Adams et al. 2006). The frequency of large rearrangements in the *BRCA1* gene is higher than in the *BRCA2* gene (Sluiter and van Rensburg 2010). The majority of large rearrangements described so far involve deletions in both genes, but the amplifications are very infrequent (Sluiter and van Rensburg 2010). Some duplications in *BRCA1* have been reported in different populations, such as the duplication of exon 13 and exon 3–8 in French patients, triplicate amplification of exons 17–19 in Dutch patients, the duplication of exon 20 in Italian patients, and duplications of exons 18 and 19 in North Americans (Puget et al. 1999; Gad et al. 2001; Hogervorst et al. 2003; Agata et al. 2006; Walsh et al. 2006). In South America, there are few reports of studies of the *BRCA* genes by MLPA, and a frequency of 3.8% in a Chilean study and 6.4% in Colombia of large rearrangements in *BRCA1* was found, whereas no alterations were found at the *BRCA2* level (Sanchez et al. 2011; Silva et al. 2014). Although our sample of patients was small (*N* = 18), the frequency of large rearrangements was 12.5% (2 of 18), similar to that reported for other populations; however, we must be careful because the small sample. Finally, there are cases where the pathogenic role of some duplications identified in *BRCA* cannot be explained; thus, it is necessary to perform segregation studies in these families to determine their possible pathogenic effect on the development of the disease (Agata et al. 2006). Among our patients, we found two independent events of multiplication of exon 7 in probands that could not be found in other cancer affected relatives, thus concluding that they are not pathogenic.

In conclusion, our comprehensive evaluation of *BRCA1/BRCA2* in families with criteria for hereditary breast cancer found three known *BRCA1/BRCA2* mutations and *BRCA1* c.4647_4648dupAA as a novel pathogenic. The alteration rate in *BRCA1/BRCA2* with proven pathogenic mutation was 22.2% (4 of 18), and this frequency can be explained by the genetic testing criteria, the reduced sample size, or a possible distinctive genetic profile of the Peruvian samples. A few VUS have also been found in our population and they need to be analyzed for its potential pathogenic effect. This report is relevant for the local population and justifies the development of other studies with a large cohort to determine the spectrum and prevalence of *BRCA1* and *BRCA2* mutations and possible founder mutations in the Peruvian population.

## Conflict of Interest

The authors declare no conflict of interest.
